# Tissue-engineered cardiac patch seeded with human induced pluripotent stem cell derived cardiomyocytes promoted the regeneration of host cardiomyocytes in a rat model

**DOI:** 10.1186/s13019-016-0559-z

**Published:** 2016-12-01

**Authors:** Tadahisa Sugiura, Narutoshi Hibino, Christopher K. Breuer, Toshiharu Shinoka

**Affiliations:** 1 Tissue Engineering Program and Surgical Research, Nationwide Children’s Hospital, Columbus, OH USA; 2 Department of Cardiothoracic Surgery, The Heart Center, Nationwide Children’s Hospital, Columbus, OH USA; 3 Cardiovascular Tissue Engineering Program, Department of Cardiothoracic Surgery, The Heart Center, Nationwide Children’s Hospital, 700 Children’s Drive, T2294, Columbus, OH 43205 USA

**Keywords:** Tissue engineering, Induced pluripotent stem cell derived cardiomyocytes, Biodegradable cardiac patch, Congenital heart disease

## Abstract

**Background:**

Thousands of babies are born with congenital heart defects that require surgical repair involving a prosthetic implant. Lack of growth in prosthetic grafts is especially detrimental in pediatric surgery. Cell seeded biodegradable tissue engineered grafts are a novel solution to this problem. The purpose of the present study is to evaluate the feasibility of seeding human induced pluripotent stem cell derived cardiomyocytes (hiPS-CMs) onto a biodegradable cardiac patch.

**Methods:**

The hiPS-CMs were cultured on a biodegradable patch composed of a polyglycolic acid (PGA) and a 50:50 poly (l-lactic-co-ε-caprolactone) copolymer (PLCL) for 1 week. Male athymic rats were randomly divided into 2 groups of 10 animals each: 1. hiPS-CM seeded group, and 2. Unseeded group. After culture, the cardiac patch was implanted to repair a defect with a diameter of 2 mm created in the right ventricular outflow tract (RVOT) wall. Hearts were explanted at 4 (*n* = 2), 8 (*n* = 2), and 16 (*n* = 6) weeks after patch implantation. Explanted patches were assessed immunohistochemically.

**Results:**

Seeded patch explants did not stain positive for α-actinin (marker of cardiomyocytes) at the 4 week time point, suggesting that the cultured hiPS-CMs evacuated the patch in the early phase of tissue remodeling. However, after 16 weeks implantation, the area fraction of positively stained α-actinin cells was significantly higher in the seeded group than in the unseeded group (Seeded group: 6.1 ± 2.8% vs. Unseeded group: 0.95 ± 0.50%, *p* = 0.004), suggesting cell seeding promoted regenerative proliferation of host cardiomyocytes.

**Conclusions:**

Seeded hiPS-CMs were not present in the patch after 4 weeks. However, we surmise that they influenced the regeneration of host cardiomyocytes via a paracrine mechanism. Tissue-engineered hiPS-CMs seeded cardiac patches warrant further investigation for use in the repair of congenital heart diseases.

## Background

Approximately 10,000 children undergo reconstructive operations to repair complex congenital abnormalities each year [1]. Unfortunately, many pediatric patients outgrow their implants much like they outgrow their shoes. In pediatric cardiac surgery, currently used prosthetic materials lack growth potential and they necessitate staged repairs or re-operations. These additional surgical procedures add additional morbidity because of increased complexity due to the formation of significant pericardial adhesions. Tissue engineering has emerged to solve this issue by creating a living graft with growth potential.

Typically a tissue-engineered graft is made up of a scaffold and seeded cells. As the scaffold degrades, neotissue forms and a living, biocompatible tissue is created. Using the classical tissue engineering paradigm, many cell types have been considered as possibilities for seeding onto a biodegradable scaffold, which provides sites for cell attachment and space for neotissue formation [2]. Induced pluripotent stem (iPS) cells were first generated by nuclear reprogramming of mouse fibroblasts in 2006 [3], and human iPS (hiPS) cells were established in 2007 [4, 5]. Recent studies have demonstrated various methods for the highly efficient production of cardiomyocytes derived from hiPS cells that maintained their typical electrophysiological functions [6]. Human iPS cells represent an unlimited source of cardiomyocytes because of their great potential for differentiation and are therefore one of the most promising sources of cells for cardiac regeneration therapy [7, 8].

While hiPS cell derived cardiomyocytes (hiPS-CMs) have been used for cardiac regeneration therapy, there are currently significant limitations, which include a very low percentage of engraftment success and cell survival. Insufficient blood and oxygen delivery is one of the most important causes of poor engraftment. To address this challenge, we will test the feasibility of a cardiac patch, seeded with hiPS-CMs on a biodegradable scaffold composed of a polyglycolic acid (PGA) and a 50:50 poly (l-lactic-co-ε-caprolactone) copolymer (PLCL), to grow into neo-tissue. This cardiac patch was implanted onto a right ventricular outflow tract (RVOT) wall defect created in an immunocompromised rat heart, so that the patch will receive blood supply directly from the luminal surface. The purpose of this study is to demonstrate the feasibility and application of this tissue-engineered patch in the repair of a cardiac defect using rat RVOT reconstruction model.

## Methods

### Cell culture

The hiPS-CMs were obtained commercially (Cellular Dynamics International (CDI), Madison, Wisconsin). To perform immunofluorescent staining the hiPS-CMs were cultured on a 4 well chamber mounted on glass slides with a cover (Chember Slide System; nunc, NY, USA) at a density of 1.0 × 10^5^ cells for 1 week. Cell culture media (iCell Cardiomyocytes Maintenance Medium: CDI) was changed every 2 days. The hiPS-CMs were labeled with a red fluorescent protein, which allowed us to track the engraftment of the cells.

### Preparation of tissue-engineered cardiac patch

A scaffold composed of a woven fabric of polyglycolic acid (PGA) and a 50:50 poly(l-lactic-co-ε-caprolactone) copolymer (PLCL) was constructed as previously described [9]. The scaffold is more than 80% porous and 0.6 to 0.7 mm in thickness. The hiPS-CMs were cultured on the biodegradable patch with a diameter of 6 mm at a density of 2.0 × 10^5^ cells for 1 week. Cell culture media was changed every 2 days.

### Animal model and surgical implantation

All animals received humane care in compliance with the National Institutes of Health (NIH) guideline for the Care and Use of Laboratory Animals. The Institutional Animal Care and Use Committee at Nationwide Children’s Hospital approved the use of animals and all procedures described in this study. Nude athymic rats were purchased from Jackson Laboratories (Bar Harbor, ME) and used for all experiments in the present study.

Adult male nude athymic rats weighing 230–300 g were used for the right ventricular outflow tract (RVOT) reconstruction procedure as previously described [10]. Briefly, anesthesia was obtained with ketamine (50 mg/kg, i.p) and xylazine (5 mg/kg, i.p); it was maintained using isoflurane (1.5%) in oxygen. The animals were intubated with a 16-gauge catheter and respiration was maintained at 60 cycles/min with a tidal volume of 2.5 ml. The surgical procedure was performed using aseptic techniques with sterile instruments. First, the skin of the chest was sterilized with a povidone-iodine solution and the heart was exposed through a median sternotomy. A purse string suture, with a diameter slightly larger than 6.0 mm, was placed in the free wall of the RVOT with Prolene 7-0 polypropylene sutures (Ethicon, Somerville, NJ, USA). Both ends of the suture were passed through a 22-gauge plastic vascular cannulae, which was used as a tourniquet. The tourniquet was tightened and the distended part of the RVOT wall inside the purse string suture was resected. To indicate that a transluminal defect had been created in the RVOT, the tourniquet was briefly released to determine whether massive bleeding occurred. Next, the cardiac patch was sutured along the margin of the purse string suture with 7-0 polypropylene over-and-over sutures to cover the defect in the RVOT. After completion of suturing, the tourniquet was released and the purse string suture was removed. Fibrin glue (Evicel; Ethicon) was applied to the patch as well as the suture line. After expanding the lungs using positive end-expiratory pressure, the sternum was closed parasternally with four interrupted Prolene 5-0 polypropylene sutures (Ethicon). The muscle layer and skin were closed with Vicryl 4-0 absorbable sutures (Ethicon). The first 3 days after surgery, buprenorphine (0.05 mg/kg) analgesia and cefuroxime (100 mg/kg) antibiotic were administered twice a day subcutaneously. Animals were randomly divided into 2 groups of 10 animals each: 1. hiPS-CM seeded group, and 2. Unseeded group. Two animals in each group were sacrificed at 4 and 8 weeks postsurgery and 6 animals in each group were sacrificed at 16 weeks postsurgery.

### Histology and immunofluorescence

Explanted cardiac patches were fixed in 4% para-formaldehyde, embedded in paraffin, sliced (5 μm thick sections), and stained with Hematoxylin and Eosin (H&E). H&E staining was used for cell counting. One representative section from each explant at 16 weeks after implantation was stained and imaged. All positively stained nuclei were counted from high magnification images. Collagen deposition was assessed with Picrosirius red staining and images were obtained with polarized light microscopy. Based on previous reports, we correlated thick fibers (orange to yellow) with collagen I and thin fibers (green) with collagen III [11].

Immunofluorescent staining for α-actinin as a marker of cardiomyocytes was performed using rabbit anti-α-actinin (sarcomeric) primary antibody (1:200, Abcam, Cambridge, MA) followed by Alexa Fluor 488 anti-rabbit IgG secondary antibody (1:300, Invitrogen, Carlsbad, CA). Fluorescent images were obtained with an Olympus IX51 microscope (Olympus, Tokyo, Japan).

### Echocardiographic analysis

Echocardiographic measurements were obtained preoperatively and at 8 and 16 weeks postoperatively. Animals underwent isoflurane anesthesia (2% isoflurane with 100% oxygen gas inhalation through a nose cone). When the anesthesia plane was established, B-mode and M-mode echocardiography was performed (Vevo Visualsonics 770; Visualsonics, Toronto, ON, Canada). We visualized left ventricle (LV) short axis view and right ventricle (RV) outflow short axis view. RV and LV minimum and maximum diameters were measured using M-mode echocardiography and LV ejection fraction was calculated.

### Statistical analysis

Numeric values are listed as mean ± standard deviation. The number of experiments is shown in each case. Data of continuous variables with normal distribution were evaluated by student *t* test. *P* values less than 0.05 indicated statistical significance.

## Results

### Cell culture

**Fig. 1:**
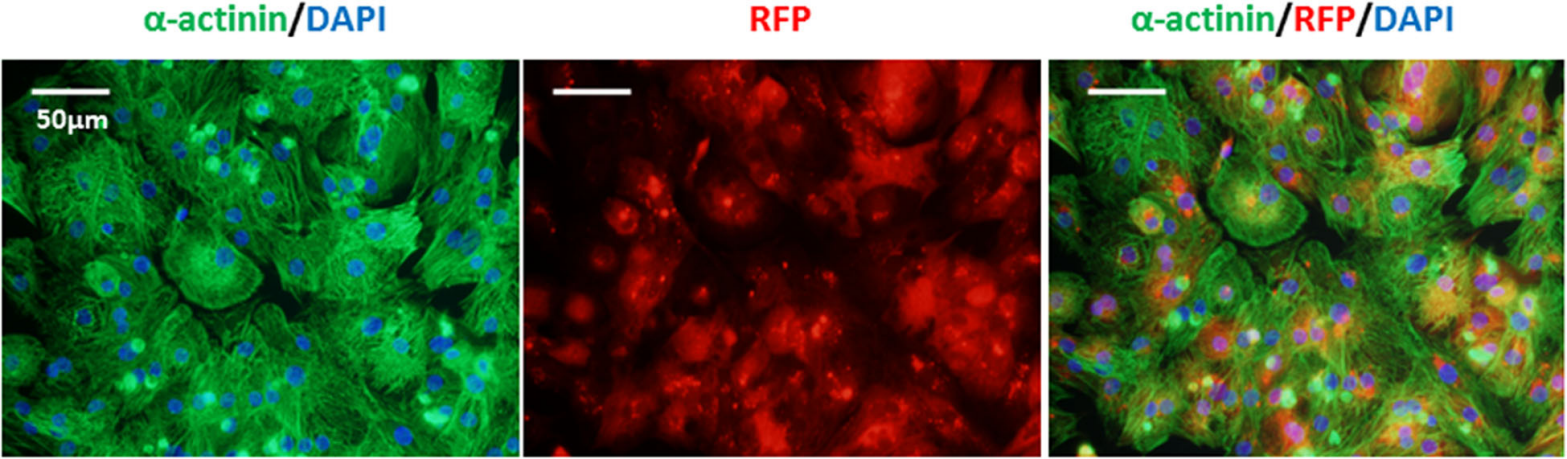
Immunofluorescent images of hiPS-CMs. Representative immunofluorescent images of α-actinin merged with red fluorescent protein that is originally expressed in hiPS-CMs and DAPI after 2 days in culture in a well

Two days after culturing, hiPS-CMs started contracting (Data not shown). Immunofluorescent staining for α-actinin showed the hiPS-CMs were positive for α-actinin and they were also positive for red fluorescent protein (Fig. 1).

### Surgical observations

**Fig. 2:**
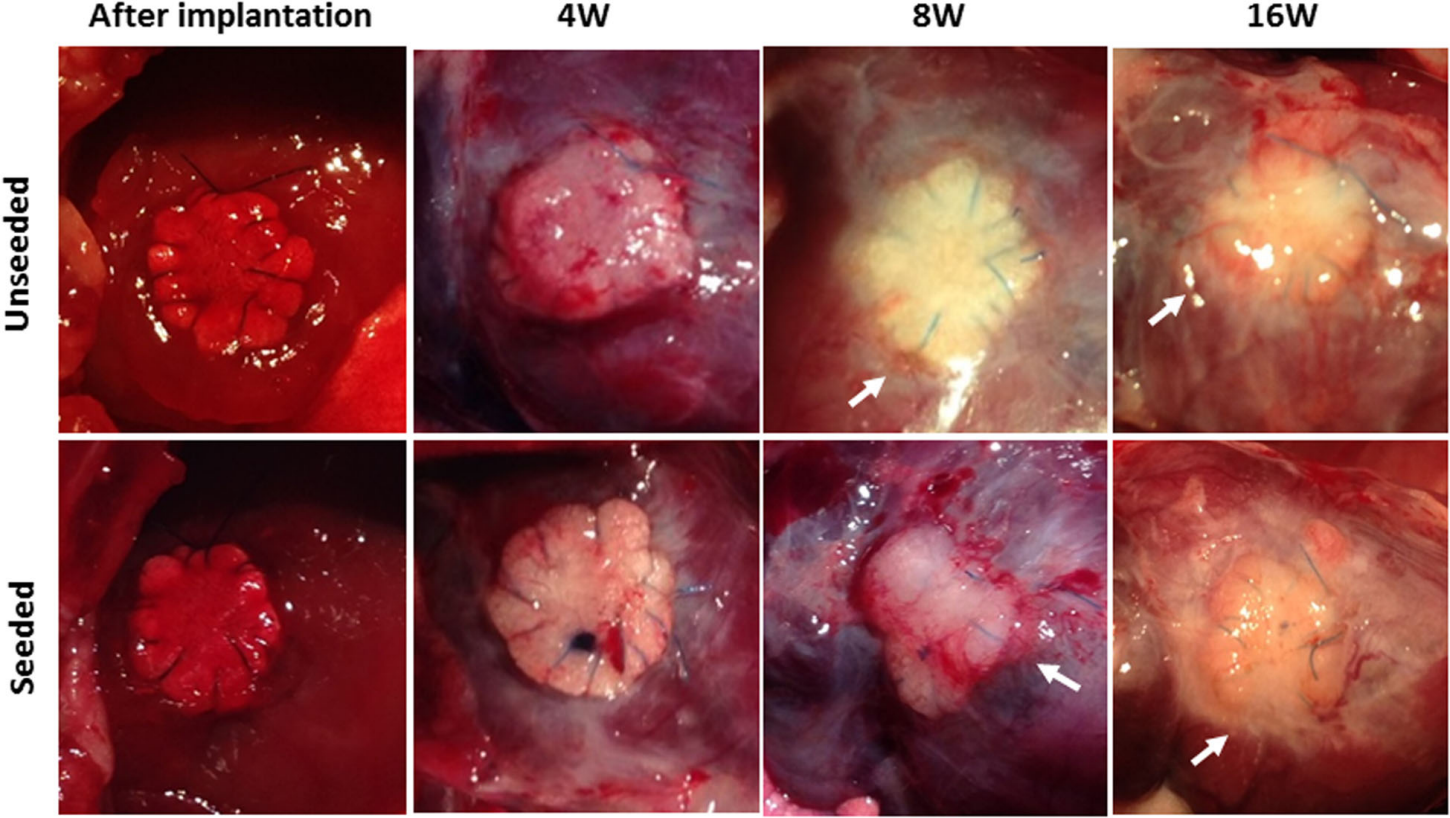
Macroscopic images of the tissue-engineered hiPS-CMs seeded or unseeded cardiac patches. There were fibrous adhesions on the epicardial surface of the patches over the course of 16 weeks in both groups (*arrows* indicate fibrous adhesions). There was no significant difference in macroscopic findings between the groups

In both groups macroscopic post implantation images of the cardiac patches showed fibrous adhesions on the epicardial surface of the patches over the course of 16 weeks (Fig. 2). There was no significant difference in macroscopic findings between the groups.

### Histology, immunofluorescent analysis

**Fig. 3:**
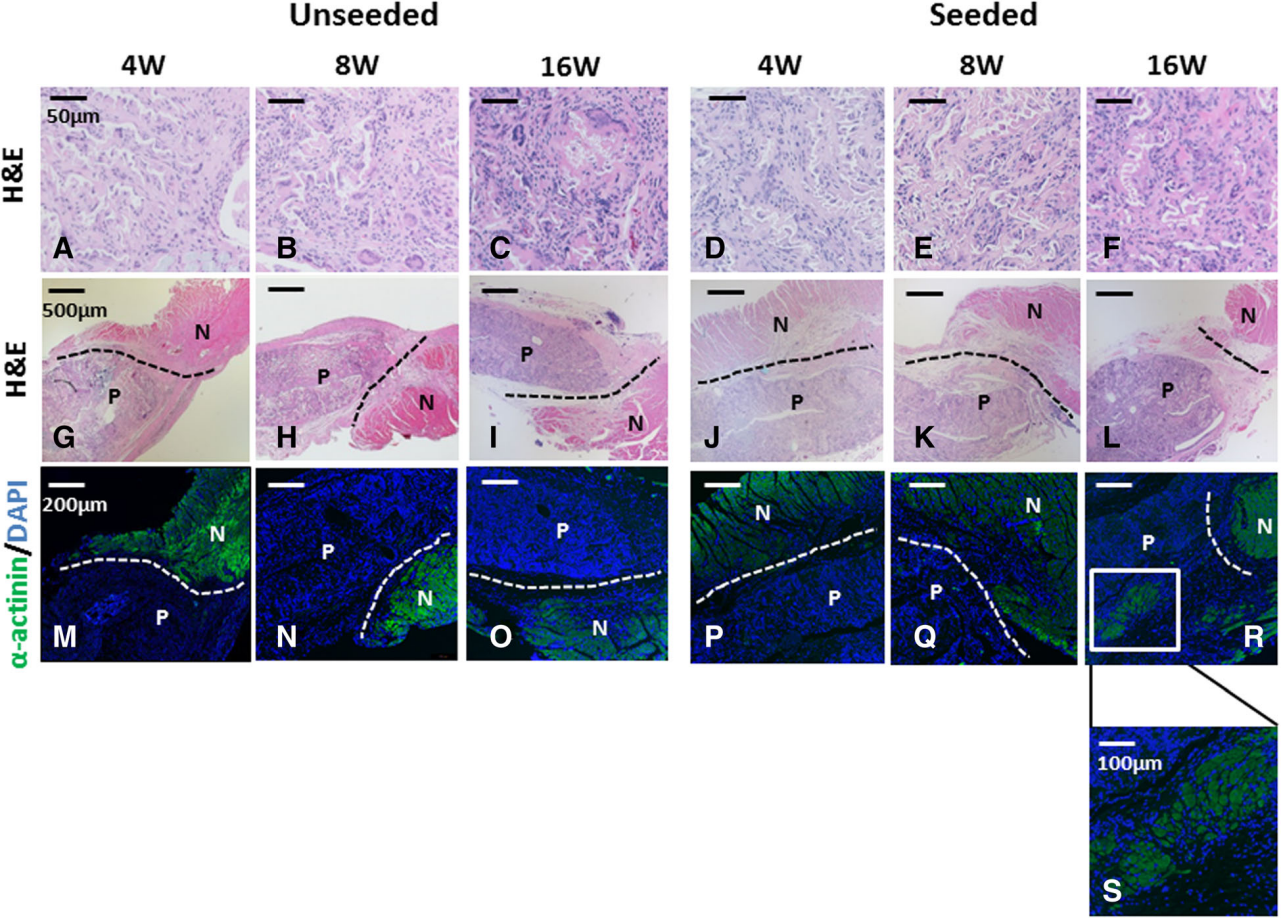
Histological analysis of the grafts at 4, 8 and, 16 weeks after implantation. Hematoxylin and Eosin (H&E) staining demonstrated dense cellular infiltration into the hiPS-CMs seeded or unseeded cardiac patches (**a** - **f**: high magnification images, **g - l**: low magnification images). Both seeded and unseeded patches explants did not stain positive for α-actinin (*green*) at the 4 and 8 week time point (**m**, **n**, **p**, **q**). However, there were small islands of cells which stained positive for α-actinin (*green*) in the cardiac patch 16 weeks after implantation (**o**, **r**, **s**). *N* native heart muscle, *P* patch

**Fig. 4:**
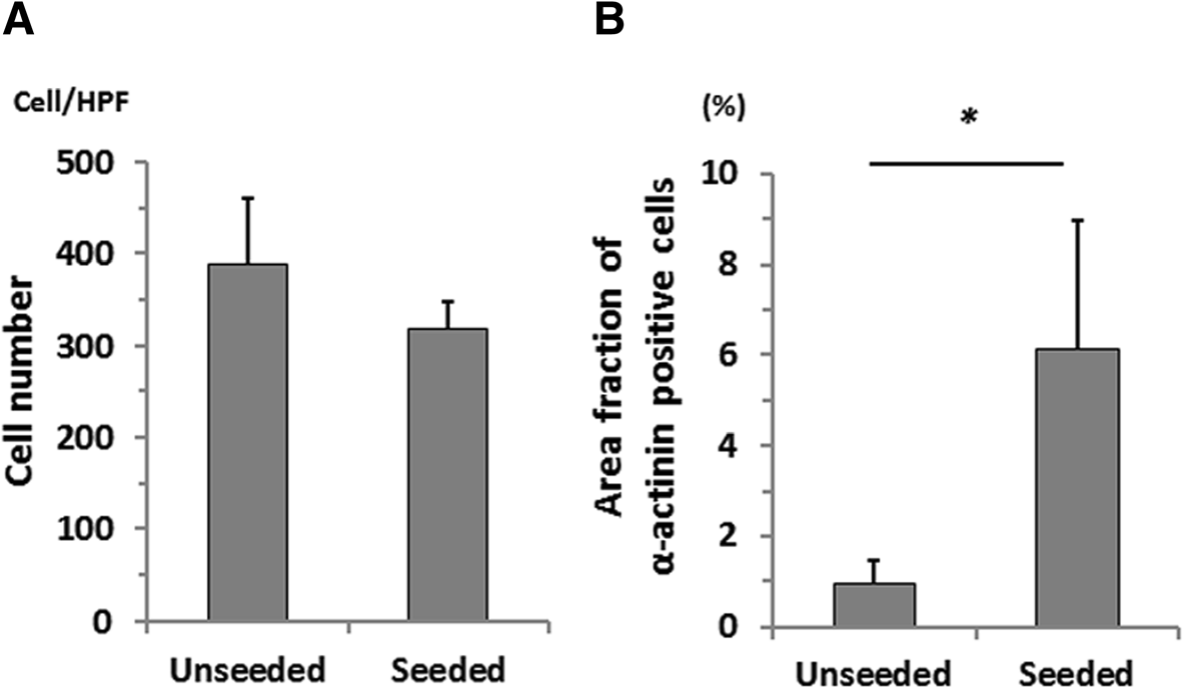
Quantitative comparison of the cellular infiltration into the scaffold and α-actinin positive cell. **a** There was no statistical difference between the groups in the cell number within the scaffolds at 16 weeks after implantation. **b** The area fraction of positively stained α-actinin cells was significantly higher in the seeded group than in the unseeded group. *: *p* < 0.05

H&E staining showed cell infiltration within the scaffold in both groups (Fig. 3), and nuclei were counted to obtain the number of cells in the scaffold. There was no statistical difference in the cell number between the groups at 16 weeks after implantation (Unseeded group: 390 ± 71/HPF vs. Seeded group: 319 ± 30/HPF, *p* = 0.08 (HPF: high power field)) (Fig. 4a).

To evaluate the engraftment of implanted hiPS-CMs, α-actinin staining was employed. Seeded patch explants did not stain positive for α-actinin at the 4 and 8 week time point, suggesting that the cultured hiPS-CMs evacuated the patch in the early phase of tissue remodeling. However, there were small islands of cells which stained positive for α-actinin in the cardiac patch 16 weeks after implantation. The area fraction of positively stained α-actinin cells was significantly higher in the seeded group than in the unseeded group (Seeded group: 6.1 ± 2.8% vs. Unseeded group: 0.95 ± 0.50%, *p* = 0.004), suggesting cell seeding promoted regenerative proliferation of host cardiomyocytes (Fig. 4b).

**Fig. 5:**
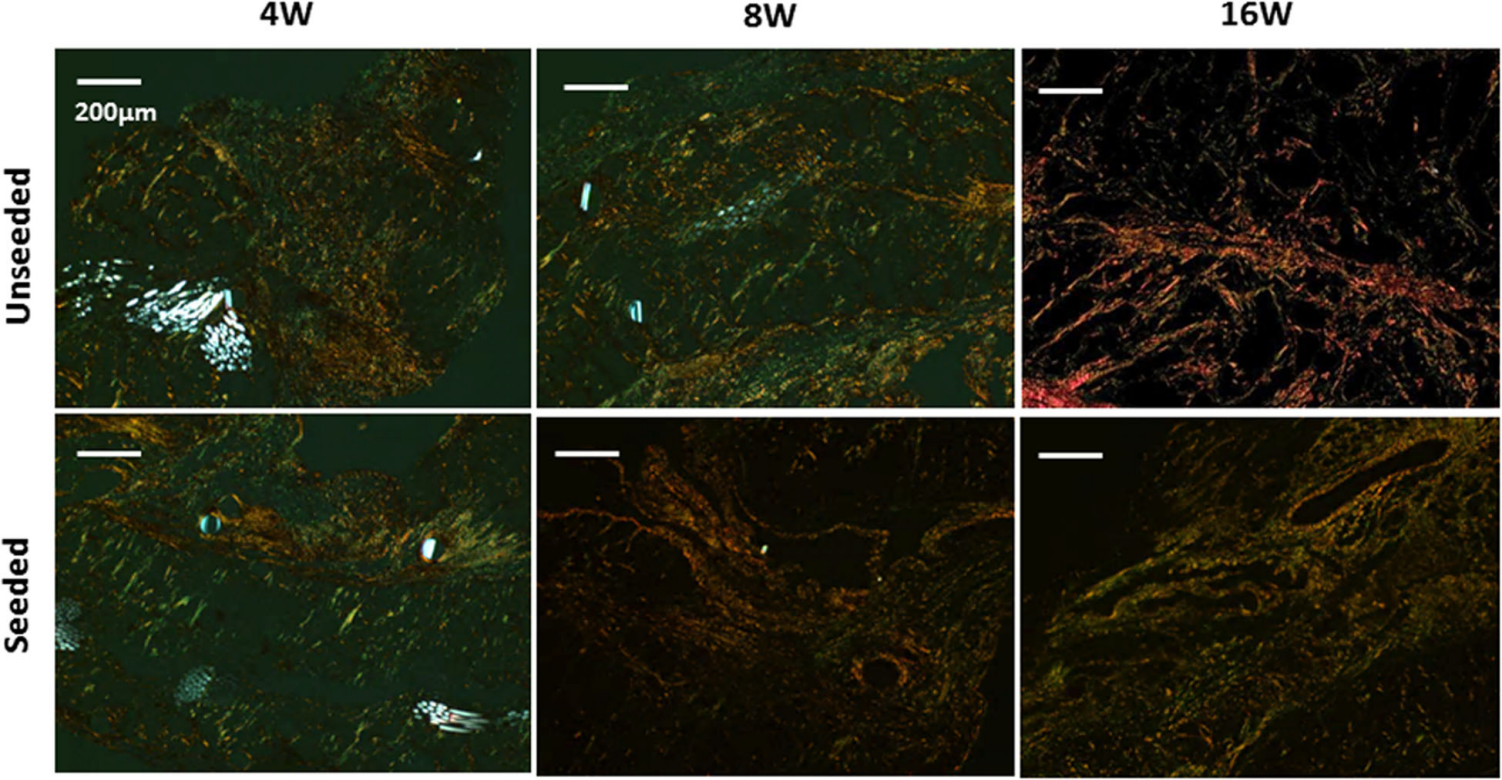
Collagen deposition in the grafts at 4, 8 and, 16 weeks after implantation. Visualization of Picrosirius red staining with polarized light microscopy showed thin (type III; *green*) to thick (type I; *yellow*) collagen fibers and scaffold fragments (*white*) in both groups. Over the course of 16 weeks, the cardiac patch gradually degraded and remodeled into collagenous tissue in both the seeded and unseeded groups

Visualization of Picrosirius red staining with polarized light microscopy shows thick orange fibers and thin green fibers which are correlated with collagen type I and type III, respectively in both groups equally (Fig. 5). Over the course of 16 weeks, the cardiac patch gradually degraded and remodeled into collagenous tissue in both the seeded and unseeded groups.

### Echocardiographic assessment

**Fig. 6:**
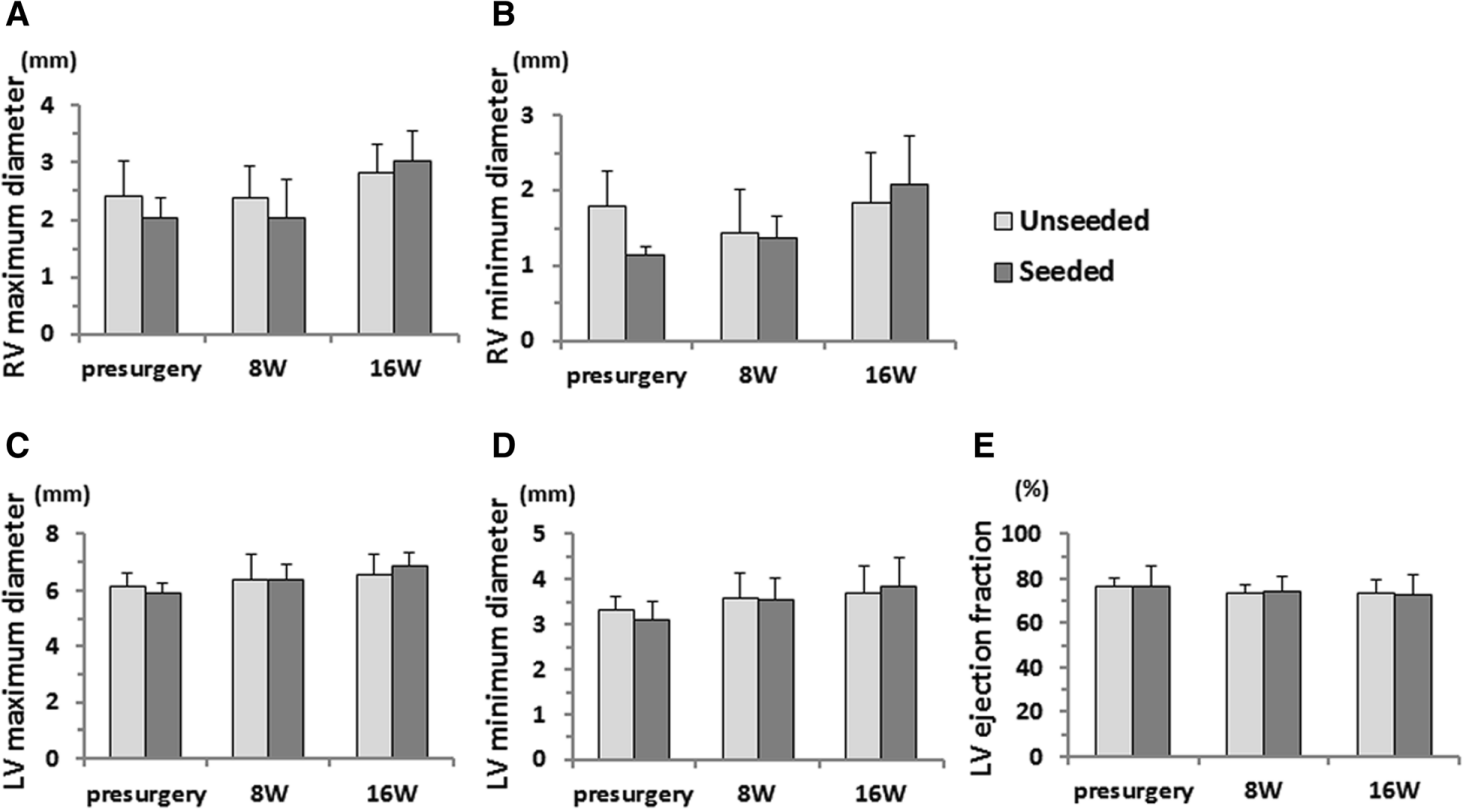
Echocardiographic analysis presurgery and 8 and 16 weeks postsurgery. **a** RV maximum diameter. **b** RV minimum diameter. **c** LV maximum diameter. **d** LV minimum diameter. **e** LV ejection fraction. There was no statistical difference in RV maximum and minimum diameters between the groups at each time point. There was no aneurysmal change in either group. There was no statistical difference in LV maximum and minimum diameters and LV ejection fraction between the groups at each time point

There was no statistical difference in RV maximum and minimum diameters between the groups at each time point (Fig. 6a, b). There was no aneurysmal change in either group. There was no statistical difference between the groups in LV maximum and minimum diameters and LV ejection fraction at each time point (Fig. 6c, d, e). Either unseeded or seeded cardiac patch implanted hearts showed no functional or dimensional dysfunction at each time point.

## Discussion

Tissue-engineering in conjunction with therapeutic therapy, is a novel approach for reconstruction of cardiac defects. Many researchers believe that paracrine effects are the major mechanism responsible for the therapeutic efficacy of stem cell or progenitor cell therapy. These effects classically refer to the ability of transplanted cells to release various cardioprotective factors into damaged cardiac tissue for attenuation of the remodeling process; in contrast, recent reports suggest that cell transplantation upregulates various cardioprotective factors in native cardiac tissue through “crosstalk” between transplanted cells [12, 13]. In addition, our group previously demonstrated, in a mouse model, that bone marrow mononuclear cells seeded onto vascular grafts disappeared in the early phase, but their initial presence mediated for the appropriate vascular remodeling and development via a paracrine mechanism [14]. In the present study, seeded hiPS-CMs were not present in the patch after 4 weeks. However, α-actinin positive cells were significantly greater in the seeded group than in the unseeded group 16 weeks after implantation. Therefore, we surmise that seeded hiPS-CMs might influence the regeneration of host cardiomyocytes via a paracrine mechanism.

Many studies performed on cardiac regeneration therapy use stem cells such as hiPS-CM sheets [15, 16] or stem cell injections [17]. However, one of the most critical issues for those studies is an insufficient blood and oxygen supply resulting in poor engraftment of transplanted hiPS-CMs. In this study, we applied a RVOT reconstruction method to implant the hiPS-CM seeded cardiac patches in conjunction with incorporating a sufficient blood supply directly from the luminal surface of the patch. In the tissue-engineering paradigm, the scaffold provides a source of cell attachment and initial structural integrity [2]. Our PGA-PLCL patch is porous and has a sponge like structure so that the seeded hiPS-CMs can grow inside the patch, which ensures adequate perfusion of nutrients and oxygen during culture. Sufficient nutrient and oxygen delivery via functional vasculature or perfusion is vital for the success of cardiac stem cell therapy.

The mammalian heart’s capacity for self-renewal is actively debated [18, 19]. Some studies suggest that new cardiomyocytes regenerate at a very low rate [20–22] and that they may be derived from the division of pre-existing cardiomyocytes in the mammalian heart. Other studies suggest a high rate of stem cell activity with continuous differentiation of progenitors to cardiomyocytes [23].

There are some limitations in this study. First, structural and functional immaturity of hiPS-CMs may result in their death in the in vivo environment. Second, the evacuation of the seeded hiPS-CMs may be due to the interspecies difference. Finally, even though we showed the cells which stained positive for α-actinin in the cardiac patch 16 weeks after implantation, we were unable to prove a mechanism for the regeneration of cardiomyocytes.

## Conclusions

In this study, the seeded hiPS-CMs were not present in the patch in the early time point. However, they might influence the regeneration of host cardiomyocytes. Tissue-engineered hiPS-CMs seeded biodegradable cardiac patches warrant further investigation for use in the repair of heart diseases.

## Abbreviations

DAPI: 4′,6-diamidino-2-phenylindole
H&E: Hematoxylin and Eosin
hiPS: Human induced pluripotent stem
hiPS-CMs: Human induced pluripotent stem cell derived cardiomyocytes
HPF: High power field
iPS: Induced pluripotent stem
LV: Left ventricle
NIH: National Institutes of Health
PGA: Polyglycolic acid
PLCL: Poly (l-lactic-co-ε-caprolactone)
RV: Right ventricle
RVOT: Right ventricular outflow tract
